# Comparative Evaluation and Physicochemical Characterisation of Three Tolerant Interspecific Grape Cultivars

**DOI:** 10.3390/plants15111663

**Published:** 2026-05-28

**Authors:** Maja Mikulic-Petkovsek, Tatjana Jovanović-Cvetković, Saša Krošelj, Rada Grbić, Dino Hasanagić, Miljan Cvetković, Denis Rusjan

**Affiliations:** 1 Biotechnical Faculty, Department of Agronomy, Chair for Fruit Growing, Viticulture and Vegetable Growing, University of Ljubljana, Jamnikarjeva 101, SI-1000 Ljubljana, Slovenia; denis.rusjan@bf.uni-lj.si; 2 Faculty of Agriculture, University of Banja Luka, 78000 Banja Luka, Republic of Srpska, Bosnia and Herzegovina; tatjana.jovanovic-cvetkovic@agro.unibl.org (T.J.-C.); rada.grbic@agro.unibl.org (R.G.); miljan.cvetkovic@agro.unibl.org (M.C.); 3 Agricultural Institute of Slovenia, Hacquetova Ulica 17, SI-1000 Ljubljana, Slovenia; sasa.kroselj@kis.si; 4 Faculty of Natural Sciences and Mathematics, University of Banja Luka, 78000 Banja Luka, Republic of Srpska, Bosnia and Herzegovina; dino.hasanagic@pmf.unibl.org

**Keywords:** tolerant grape cultivars, phenolic compounds, sugars, organic acids, berry quality parameters

## Abstract

In the study, the morphological and structural properties of bunches and berries, as well as the chemical characterisation of three interspecific grapevine cultivars—‘Bronner’, ‘Muscaris’, and ‘Morava’—were studied. Sugars and organic acids in the grapes were analysed using high-performance liquid chromatography (HPLC), while phenolic compounds were analysed by HPLC–mass spectrometry. ‘Morava’ and ‘Muscaris’ showed higher Hue angle values, indicating a greener skin coloration compared with ‘Bronner’, which exhibited a more yellow skin colour. ‘Muscaris’ and ‘Bronner’ had higher bunch weights than ‘Morava’, while the 100-berry weight did not differ significantly among the varieties. ‘Bronner’ must had the highest titratable acidity, and ‘Muscaris’ had the highest sugar content. Flavanols were the main phenolic compounds in the grapes, accounting for 76–88% of the total phenolic content. The highest concentrations of flavanols and caftaric acid were found in ‘Bronner’ and ‘Muscaris’ berries, while ‘Morava’ had the highest flavonol content. All studied varieties achieved good grape and must quality and are therefore recommended for wine production. ‘Bronner’ and ‘Muscaris’ stood out for their high bunch weight and high concentrations of flavanols and total phenolics, which contribute significantly to wine taste and overall quality. ‘Muscaris’ berries had a high sugar content, making this variety suitable for producing wines with higher alcohol content. In contrast, ‘Bronner’ is recommended for wines requiring higher acidity levels.

## 1. Introduction

In traditional and especially in European vitiviniculture, it has long been believed that only European grapevine cultivars (*Vitis vinifera* L.) can produce high-quality wines. However, these cultivars are generally more susceptible to diseases (downy mildew, powdery mildew) and pests (Phylloxera) than other *Vitis* species. As a result, these vines require regular protection with phytopharmaceutical products (PPPs), leading to high production costs as growers must use these expensive products. This also has a negative environmental impact [1,2].

Contemporary grape and wine production is undergoing significant changes due to formal requirements (policies) related to environmental protection and human health. To ensure long-term sustainability, it is essential to preserve natural resources (climate, soil, water) through ecologically sustainable practices [3]. The European Union’s plans and the 2050 Green Deal require drastic changes in agricultural production, primarily to reduce the use of chemical agents [4]. The European Union’s “Farm to Fork Strategy” aims to reduce the use and risks of chemical pesticides by 50% across Europe by 2030 [5].

The introduction and use of disease-tolerant grapevine cultivars in vineyards would therefore be a long-term and highly suitable measure for controlling downy mildew, powdery mildew and grey mould, mostly [6] or some bacterial diseases, e.g., Petrie’s disease [7]. One approach would be to increase the proportion of interspecific hybrids in total grape and wine production, as these are more tolerant to certain diseases. Disease-tolerant grapevine cultivars are obtained by crossing *V. vinifera* cultivars with American native grapevine species, or by crossing hybrids with *V. vinifera* cultivars, or by crossing different hybrids [8]. Several breeding programmes have led to the development of cultivars with different characteristics, such as desirable sensory qualities, short or long growing seasons, and pest-resistance [9]. Currently, due to global climate change and harsher growing conditions such as drought, heat, and frost, most breeders are focusing their breeding programmes on developing grapevine cultivars with high resistance to diseases and other abiotic stresses through interspecific hybridisation [10].

According to the study on disease-resistant grapevine cultivars by Bavaresco, et al. [11], about 6% of the world’s viticultural area is covered by hybrids. Grapevine hybrids for wine production are mainly found in America (USA, Canada, and Brazil) and Europe (Germany, Austria, Moldova, Russia, Hungary, and Serbia). Additionally, the literature indicates that in some other Eastern European countries, interspecies hybrids also occupy a significant place in the production assortment. Germany leads with 3% (3.000 ha), followed by Austria, with 2% according to the total national viticultural area [12]. Moreover, in Bosnia and Herzegovina, the contribution of interspecific hybrids to total viticultural production remains relatively small (less than 0.5%), although Jovanović-Cvetković, et al. [13] observed an increase in certain winegrowing regions.

The quality of interspecies hybrid grapes and the market acceptance of their wine have been discussed for decades, even a century, since the first breeding programmes in their creation in the first half of the 19th century, largely due to their “foxy” and herbaceous aromas and high contents of methanol, which is harmful to human health [14,15]. The sensory profile of wines made from interspecies hybrids can differ significantly from *V. vinifera* wines due to the markedly different chemical composition of their berries [8]. Therefore, it is of primary importance for wine producers seeking to optimise winemaking protocols to assess the quality of grapes from these cultivars by examining their physicochemical characteristics.

Sugars and organic acids are among the most important compounds determining grape and wine quality, as they influence sweetness, acidity, flavour balance, microbial stability, fermentation, and sensory perception [16]. Organic acids, especially tartaric and malic acid, play a central role in maintaining wine pH, freshness, colour stability, and microbiological stability, while sugar accumulation determines sweetness and potential alcohol content during fermentation [17,18,19]. During grape ripening, the balance between sugars (mainly glucose and fructose) and acids (mainly tartaric and malic acids) is a key indicator of grape maturity and harvest timing [20].

Phenolic compounds, in addition to primary metabolites, have a significant influence on the overall quality of grapes and, consequently, on wine quality [21]. These compounds are a highly heterogeneous group that contribute to the colour and taste of grapes and wine. The most abundant class of compounds in grapes is flavan-3-ols, which include both monomeric (e.g., catechin and epicatechin) and polymeric forms (e.g., procyanidins) [22]. The polymeric forms, also known as proanthocyanidins or condensed tannins, contribute to taste by shaping mouthfeel, primarily through bitterness and astringency. They also influence the release and perception of volatile aroma compounds [22]. In grape berries, hydroxycinnamic acids such as caffeic, *p*-coumaric, and ferulic acid are mainly present as tartaric acid esters (caftaric, coutaric, and fertaric acids) [23]. In red grapes, anthocyanins are the major group and are primarily responsible for the colour intensity and stability of red wines. During vinification and ageing, they interact with tannins to form complex polymeric structures, which enhance colour stability and contribute to the sensory complexity and overall quality of aged wines [24,25]. They are also known for their antioxidant activity, as they are effective radical scavengers. Phenolics also participate in redox reactions, contributing to chemical interactions with other organic compounds. Their health benefits are important for humans, as they have antimicrobial, anticarcinogenic, and antiallergic effects, as well as reducing the risk of cardiovascular disease [26,27]. Phenolics are also important for plants, as they protect against abiotic and biotic stress factors, such as UV radiation, pathogens, and herbivores. The synthesis of these substances is strongly influenced by several factors, including environmental conditions, temperature, precipitation, soil, light, and vineyard production technology [21,28]. Phenolic profiles also differ among different grapevine species and cultivars, and can therefore be used as markers for verifying the authenticity of grapes and wine [29].

As assessing grape quality is crucial for wine producers, this study aimed to determine the quality parameters of three disease-resistant white grapevine cultivars with different parentages. Among the quality parameters of clusters and berries, cluster and berry size and weight, skin colour, the proportion of berries in the cluster, skin portion per berry, and other fruit quality parameters were monitored, as well as the content of individual sugars and organic acids, and the phenolic composition of the grapes. The results of the study will contribute to our existing knowledge of the characteristics of resistant grapevine cultivars regarding their carpometry and chemical properties. The cultivars are characterised in such detail for the first time, particularly concerning colour parameters, and a detailed analysis of phenolic compounds is carried out. The findings will inform decisions on planting these cultivars, especially considering climate change, ecological requirements in production, and the increasing demand for organically produced wine.

## 2. Results and Discussion

### 2.1. Berry Colour and Physico-Structural Characteristics

The colour of the grape berry is a key indicator of the external quality of grapes and also reflects their degree of ripeness. There were some significant differences in some colour parameters among the studied cultivars (Table 1). Significant differences were observed in parameter “b”, which is also closely related with Chroma (C*). Chroma (C*) is most commonly used to determine the degree of colour shade compared to grey. Pathare, et al. [30] state that there is a significant link between C* and fruit colour intensity. The ‘Muscaris’ cultivar had the highest Chroma value (12.61), followed by the ‘Bronner’ cultivar (9.66), while the lowest Chroma value and thus the palest, least saturated skin colour was measured in the ‘Morava’ cultivar (5.96). The hue angle (h*) represents the colour of the fruit in degrees: 0° for red, 90° for yellow, 180° for green, and 270° for blue [31]. Table 1 shows that the ‘Morava’ and ‘Muscaris’ cultivars had a significantly higher hue angle compared to the ‘Bronner’ cultivar (94.75°). This shows that the ‘Bronner’ cultivar had a slightly more yellow skin colour compared to the green-yellow skin of the ‘Morava’ and ‘Muscaris’ cultivars. From the measured colorimetric values, the CIRG index was calculated, which ranged from 1.64 to 2.04. Despite the significant differences observed in the CIRG index between the cultivars, the berry colour was still indicated to be green-yellow. The change in skin colour from green to yellow results from a decrease in chlorophyll content during grape ripening and an increase in yellow pigments in white grape cultivars, as well as an increase in anthocyanins in red grape cultivars [32].

‘Bronner’ and ‘Muscaris’ cultivars had longer clusters (15.73 cm and 16.14 cm) and therefore a higher average cluster weight than the ‘Morava’ cultivar (13.76 cm) (Table 1). Cluster weight is certainly the most important factor for growers. The cluster weight for ‘Bronner’ was 281.5 g and for ‘Muscaris’ 254 g, both higher than ‘Morava’, which had an average cluster weight of 177.5 g. The latter value was significantly lower, with Ivanišević, et al. [33] reporting a cluster weight for ‘Morava’ of 232 g from conventional production. The ‘Morava’ cultivar is very suitable for organic production, despite a slightly lower yield per vine, as shown by Ivanišević, Kalajdžić, Cindrić, Korać and Božović [33]. The Veneto region (Italy) reports extremely low cluster weights for ‘Muscaris’ (94 g) and ‘Bronner’ (110 g) [11]. In the cultivars studied in our experiment, the mass of 100 berries ranged from 163.6 to 173.1 g, with no significant difference among the cultivars. Compared with the results of Casanova-Gascón, et al. [34], the mass of 100 berries in the ‘Muscaris’ cultivar was 31% higher in our study. The average number of seeds per berry, ranged from 2.2 in ‘Morava’ to 2.8 in ‘Bronner’. The proportion of flesh in the berries was more than 83%, the skin accounted for around 10%, and the seeds comprised approximately 6% of the total berry mass (Table 1).

### 2.2. Assessment of Chemical Quality of Grapes

The measured total soluble solids (TSS) for the studied cultivars ranged from 22.03 to 23.55 °Brix (Table 1). The ‘Bronner’ cultivar from the Banja Luka area had a slightly higher TSS (22.32 °Brix) and total titratable acids (TTA) of 7.35 g/L in the year of the experiment compared to grapes from the Trentino, Italy location (20.19 °Brix, 7.1 g/L) [35] and the results reported from the same location by Pedò, et al. [36] (20.4 °Brix, 6.33 g/L), and a slightly lower TSS content compared to the Sao Joaquim location in Brazil (22.5 °Brix) [37]. This study also measured a higher TSS content (23.55 °Brix) in ‘Muscaris’ grapes than the values reported from sites in Wroclaw and Lublin, Poland (22 °Brix) [38,39], although the TSS was slightly lower than the result from the Trentino site (23.7 °Brix) [36].

The highest total titratable acid (TTA) content was found in the ‘Bronner’ cultivar (7.35 g/L), while the lowest TTA content was found in the ‘Muscaris’ cultivar (5.56 g/L) (Table 1), at 0.97 and 1.74 g/L less than the content reported by Pedò, Bottura and Porro [36] and Porro, Wolf and Pedò [35], and 1.66 to 1.77 g/L more than that reported by Casanova-Gascón, Ferrer-Martín, Bernad-Eustaquio, Elbaile-Mur, Ayuso-Rodríguez, Torres-Sánchez, Jarne-Casasús and Martín-Ramos [34]. Titrating acid contents are closely correlated to the pH value of the must. The cultivar with a significantly higher TTA content had a lower pH value, and vice versa. The values for the pH, TTA, and TSS of the ‘Bronner‘ cultivar (Table 1) are comparable to those reported for this cultivar in the Wädenswil area in Switzerland (TSS from 19 to 22.5 °Brix, TTA 7.3–9.6 g/L, and pH 3.1) [40]. In contrast, the TTA values obtained in an experiment in Russia [41] for the ‘Morava’ cultivar (8.9 to 11.5 g/L) were significantly higher than our TTA value for the same cultivar (5.56 g/L). The higher acid content is likely due to the harsher climate, lower temperatures, and fewer hours of sunlight in Russia compared to our location in Bosnia and Herzegovina. Similarly, during a four-year monitoring period of the ‘Morava’ cultivar in Serbia (Sremski Karlovci), slightly higher TTA values were reported, with an average TTA in must from organic production of 6.77 g/L and from conventional grape production of 7.34 g/L [33].

### 2.3. Determination of Individual Sugars and Organic Acids in Grapes

The contents of individual sugars and organic acids in the grapes of the studied cultivars were analysed. The main sugars in grapes are glucose and fructose, which account for more than 90% of the total sugar content, consistent with the findings of El Kersh, et al. [42]. Both sugars are present in grapes at an approximate ratio of 1:1. Sucrose is present in the must at a lower content (10.74 g/kg for ‘Bronner’ to 13.34 g/kg for ‘Muscaris’), comprising only 7–8% of the total sugars (Table 2). The ‘Muscaris’ cultivar had the highest content of glucose (85.15 g/kg) and fructose (83.93 g/kg), placing it first in total sugar content (182.46 g/kg). It is followed by the ‘Bronner’ cultivar (161.89 g/kg), while the ‘Morava’ cultivar had a significantly lower sugar content (153.19 g/kg). Nadulski, Sobczak, Mazur and Lysiak [39] reported a 20% higher glucose content and a 50% higher fructose content for the ‘Muscaris’ cultivar. In a study examining the effect of adding pectolytic enzymes to grape must on sugar content, it was found that the addition of these enzymes did not cause a significant increase in the sugar content of grape juice [43]. The sugar content in the must depends mainly on the cultivar used [44]. As in our study, the ‘Muscaris’ cultivar had the highest content of both monosaccharides (i.e., glucose and fructose) and total sugar content in the juice, as reported by Nadulski, Sobczak, Mazur and Lysiak [39]. According to Zubaidi, Czaplicka, Kolniak-Ostek and Nawirska-Olszanska [43], this ranged from 279.3 g/L to 290.4 g/L, which is significantly higher (1.5 to 1.6 times higher) compared to our value of total sugars in the ‘Muscaris’ cultivar.

In ‘Bronner’, ‘Muscaris’ and ‘Morava’ grapes, tartaric and malic acids comprised 85–88% of the total acid content, while citric acid accounted for 10–15% (Table 2). Fumaric and shikimic acids were present only in trace amounts. The ‘Muscaris’ cultivar had the highest malic and citric acid contents, and the ‘Bronner’ cultivar the lowest. Consequently, the total acid content was also highest in ‘Muscaris’, which had a 30% higher total acid content than ‘Bronner’. There were no differences in tartaric acid content between the cultivars. In contrast, Casanova-Gascón, Ferrer-Martín, Bernad-Eustaquio, Elbaile-Mur, Ayuso-Rodríguez, Torres-Sánchez, Jarne-Casasús and Martín-Ramos [34] reported that ‘Muscaris’ had a lower total acid content compared to the two tolerant cultivars, ‘Sauvignon Kretos’ and ‘Souvignier Gris’. Due to its lower acid content, the ‘Bronner’ cultivar also had the highest calculated sugar-to-acid ratio, indicating a sweeter taste than the ‘Morava’ and ‘Muscaris’ cultivars. The concentrations of sugars (fructose and glucose) in white wine positively influence consumer preference, whereas catechin content has a negative effect [45].

### 2.4. Determination of Phenolic Compounds in Grapes

Plants have various defence mechanisms to cope with different types of stress. One such mechanism is the synthesis of secondary metabolites, including phenolic compounds. These compounds and flavonoids exhibit antioxidant activity by donating electrons to neutralise reactive oxygen species, thereby stabilising free radicals and preventing oxidative damage to proteins, lipids, and DNA [46]. Several studies have shown that American grapevine species contain significantly higher levels of phenolic acids and flavan−3-ols than *V. vinifera*. Ruocco, et al. [47] report phenolic acid contents ranging from 18 to 133 mg/kg in the skin of *V. vinifera* and from 20 to 265 mg/kg in the berry skin of American grapevine species. For flavanols, the difference between *V. vinifera* genotypes (335 to 1148 mg/kg) and American grapevine species (*V. californica*, *V. arizonica*, *V. cinerea*) was even greater (61 to 3120 mg/kg).

The individual phenolics from different phenolic groups were analysed in grapes of the ‘Morava’, ‘Bronner’ and ‘Muscaris’ cultivars. Five representatives from the hydroxybenzoic acid group, twelve hydroxycinnamic acid derivatives, eighteen flavanols, seventeen flavonols, and nine stilbenes were identified. Information about the identified phenolic compounds and the HPLC-MSn fragmentation data in the studied interspecific grape cultivars is given in Supplementary Table S1, and the chromatograms are shown in Figures S1 and S2. The confirmation of phenolic compounds was performed according to previously published studies [48,49,50,51].

**Figure 1:**
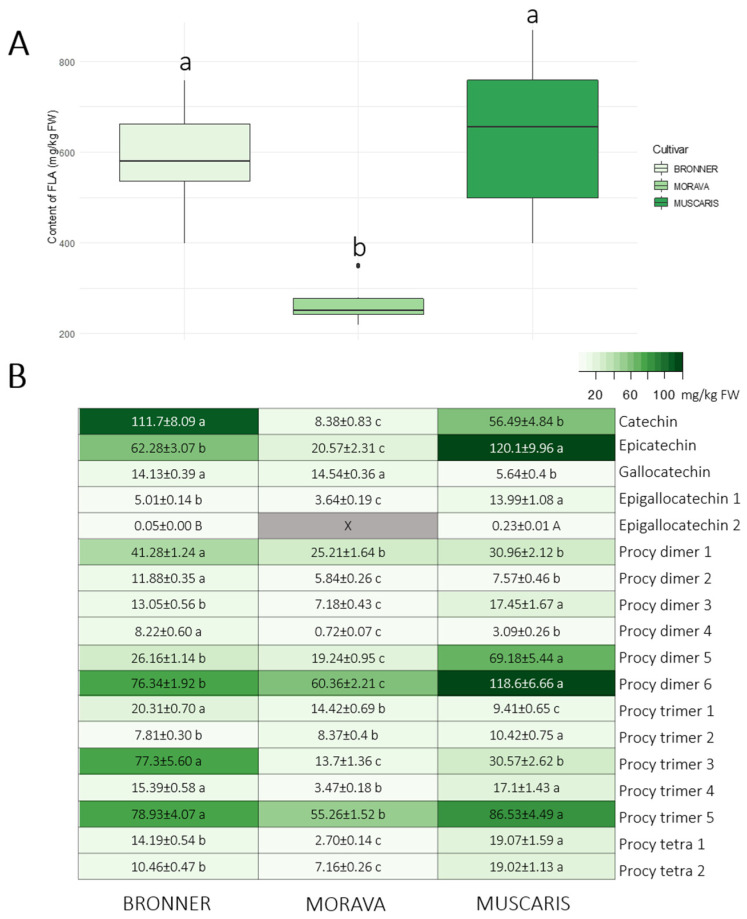
Total (**A**) and individual (**B**) flavanol (FLA) contents (mg/kg FW) in ‘Bronner’, ‘Morava’ and ‘Muscaris’ Grape Cultivars. The heatmap (**B**) is based on the average values of each individual compound, with numerical values representing the mean ± standard error. Letters indicate significant differences among cultivars, determined using one-way ANOVA and Tukey’s HSD test (lowercase letters) or LSD test (uppercase letters) at a 95% confidence interval. Abbreviations: Procy = procyanidin; tetra = tetramer.

Flavanols are predominant phenolic group due to their high contents, accounting for 76 to 88% of the total phenolic content. Previous studies also indicate that flavan-3-ols are the major group of phenolics in different grapevine cultivars [52,53]. The highest content of flavanols in grapes are found in the seeds and berry skin [53]. The cultivars richest in flavanol content are ‘Bronner’ (594.5 mg/kg FW) and ‘Muscaris’ (639.6 mg/kg FW), which have 2.2 to 2.4 times higher content compared with ‘Morava’ (Figure 1A). Gallocatechin, catechin, epicatechin, two epigallocatechins, six procyanidin dimers, five procyanidin trimers, and two procyanidin tetramers were analysed in the grapes of the studied cultivars (Figure 1B).

Similarly, Sikuten, Stambuk, Andabaka, Tomaz, Markovic, Stupic, Maletic, Kontic and Preiner [22] report that the predominant monomeric flavanol units found in grapes are catechin, gallocatechin, epicatechin, epicatechin gallate and epigallocatechin. Previous studies investigating the monomeric and oligomeric composition of flavan-3-ols in white grapes have reported the presence of several B-type dimer structures [54]. The following flavanols are notable for their high content in the ‘Muscaris’ and ‘Bronner’ cultivars: catechin, epicatechin, procyanidin dimer 5 and 6, and procyanidin trimer 5. Our catechin and epicatechin contents in the ‘Muscaris’ cultivar are comparable to those reported by Barnaba, et al. [55]. Figure 1B shows that the ‘Morava’ grape characteristically has the lowest content of almost all individual flavanols analysed. The content of flavan-3-ols in different parts of the vine depends primarily on the species or cultivar, as well as the health status of the plant. It is well established that the concentration and composition of individual phenolic compounds are strongly influenced by grapevine genotype, as well as environmental factors such as temperature, light exposure, water availability and pathogen infection. These factors influence the accumulation of phenolic compounds by regulating the expression of genes involved in the phenylpropanoid biosynthetic pathway, such as PAL, CHS, F3′H, F3′5′H and UFGT [56,57].

According to Atak, et al. [58], the catechin and epicatechin content in grapevine leaves is more dependent on health status than on cultivar. Downy mildew and powdery mildew diseases in the susceptible ‘Italia’ cultivar caused a significant increase in the content of catechin and epicatechin in the leaves compared to disease-resistant cultivars, which had lower levels of these monomeric flavanols after infection. According to Narduzzi, et al. [59], the skin of *V. vinifera* cultivars (5500 to 17,000 mg/kg) contains significantly more procyanidins than that of American grapevine species (200–650 mg/kg).

**Figure 2:**
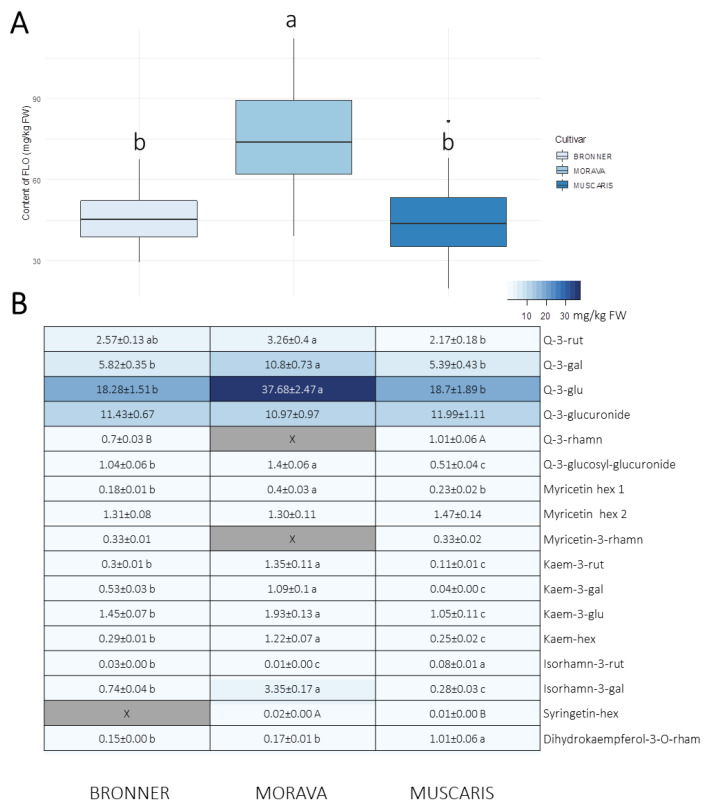
Total (**A**) and individual (**B**) flavonol (FLO) content (mg/kg FW) in ‘Bronner’, ‘Morava’ and ‘Muscaris’ Grape Cultivars. The heatmap (**B**) is based on the average values of each individual compound, with numerical values representing the mean ± standard error. Letters indicate significant differences among cultivars, determined using one-way ANOVA and Tukey’s HSD (lowercase letters) or LSD test (uppercase letters) test at a 95% confidence interval. Abbreviations: Q = quercetin; Kaem = kaempferol; Isorhamn = isorhamnetin; rhamn = rhamnoside; hex = hexoside; rut = rutinoside; gal = galactoside; glu = glucoside.

The second group with a high content is flavonols, from which six quercetin glycosides, four myricetin derivatives, five kaempferol glycosides, two isorhamnetin derivatives, and one syringetin glycoside were analysed. In contrast to the group of flavanols, where the ‘Morava’ cultivar had the lowest content, this cultivar stood out with the highest content of flavonols (75 mg/kg FW) (Figure 2A). The ‘Bronner’ and ‘Muscaris’ cultivars had a total flavonol content of only 45 mg/kg FW. Figure 2B shows that the main flavonols in grapes were quercetin-3-galactoside, quercetin-3-glucoside, and quercetin-3-glucuronide, as they represented almost 80% of the total flavonol content. Flavonols influence the taste of wine. Adding flavonols to wine increases its astringency and bitterness [60]. However, flavonol levels in wine tend to decrease over time, suggesting that long-term ageing also reduces antioxidant and colour-stabilising compounds, which may affect its sensory properties [61].

Quercetin-3-glucuronide and quercetin-3-glucoside are typical flavonol glycosides found in grapes of vines of subgenus Euvitis [62]. Other identified flavonol derivatives were present in grapes at very low contents, below 0.35 mg/kg FW. A study investigating the impact of gamma radiation on the concentration of phenolic compounds in different grape cultivars found that lower radiation levels (10 and 20 Gy) increased flavonoid content, while higher levels decreased it. This suggests a protective role for flavonoids against reactive oxygen species (ROS). The increase in phenolic compound content can be attributed to gamma radiation breaking down larger compounds into smaller ones and releasing additional phenolic compounds from their glycoside forms [63].

**Figure 3:**
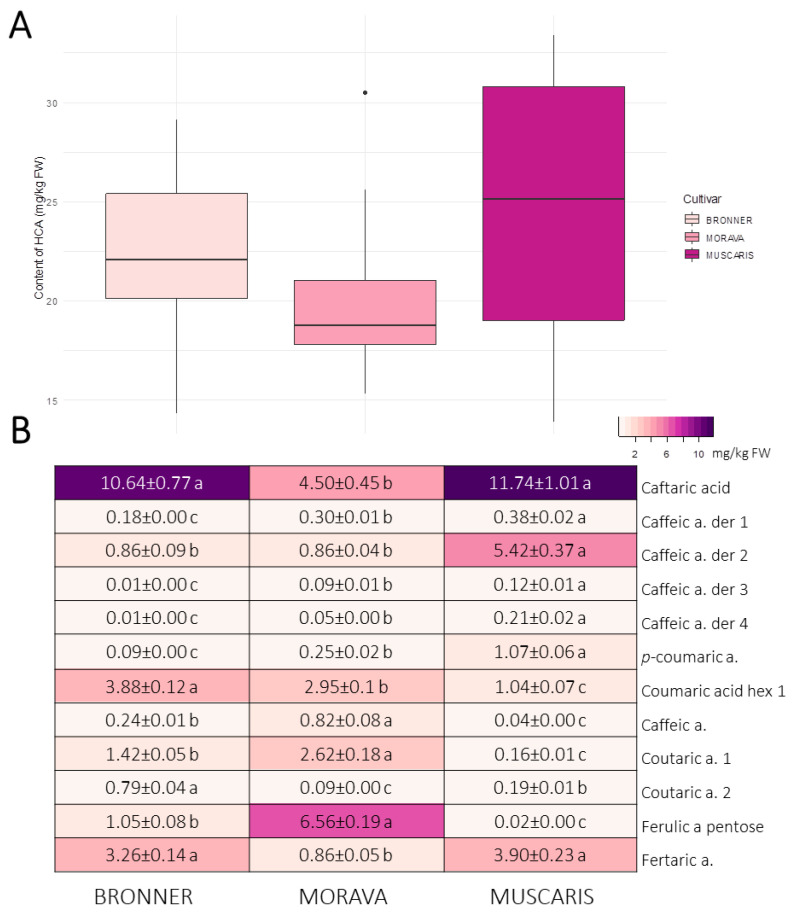
Total (**A**) and individual (**B**) hydroxycinnamic acid (HCA) content (mg/kg FW) in ‘Bronner’, ‘Morava’ and ‘Muscaris’ grape cultivars. The heatmap (**B**) is based on the average values of each individual compound, with numerical values representing the mean ± standard error. Letters indicate significant differences among cultivars, determined using one-way ANOVA and Tukey’s HSD test at a 95% confidence interval. Abbreviations: a = acid; hex = hexoside; der = derivative.

In the grapes of the studied cultivars, caftaric acid, two coutaric acids, caffeic acid, four derivatives of caffeic acid, coumaric acid derivative, fertaric acid, derivative of ferulic acid, and a derivative of p-coumaric acid were found among the hydroxycinnamic acids (HCA). Regarding high contents, caftaric acid is the most abundant, ranging from 4.5 mg/kg FW (‘Morava’) to 11.7 mg/kg FW (‘Muscaris’), followed by fertaric acid (0.86 to 3.90 mg/kg), coumaric acid hexoside (1.04 to 3.88 mg/kg), and ferulic acid pentose (0.02 to 6.56 mg/kg) (Figure 3B). Other hydroxycinnamic acids are present in grapes at lower concentrations (Figure 3B). In white grapes, hydroxycinnamic acids influence the browning process through oxidation. Among these acids, caftaric acid shows the highest browning intensity, followed by caffeic acid, while the other phenolic acids cause very low levels of browning [64]. The ‘Muscaris’ and ‘Bronner’ cultivars had the highest levels of caftaric acid and feruloyltartaric acid. Due to the high caftaric acid content in these two cultivars, as previously noted, the grape of these cultivars is more susceptible to browning. There were no significant differences in the total HCA content between the cultivars (Figure 3A). The phenolic acid contents in grapes are comparable to the results of Zubaidi, Czaplicka, Kolniak-Ostek and Nawirska-Olszanska [43], who analysed their presence in several grape cultivars. Zubaidi, Czaplicka, Kolniak-Ostek and Nawirska-Olszanska [43] found that adding pectolytic enzymes to grape pulp increases the concentration of caftaric acid in the pressed juice. This indicates that the enzyme selectively enhances the extraction of certain phenolic substances.

Stilbenes are essential defence compounds that contribute to the resistance of grapevine leaves against the major fungal pathogens *Botrytis cinerea*, *Plasmopara viticola*, and *Erysiphe necator*. Upon infection, resistant grapevine cultivars rapidly produce high levels of the toxic stilbenes δ-viniferin and pterostilbene at the sites of pathogen entry [65].

**Figure 4:**
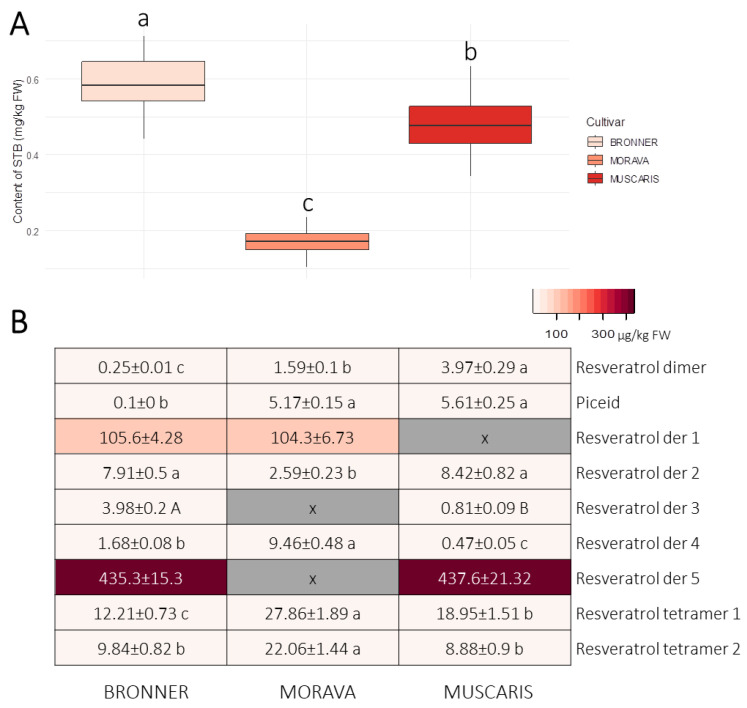
Total ((**A**); mg/kg FW) and individual ((**B**); µg/kg FW) stilbene content in ‘Bronner’, ‘Morava’ and ‘Muscaris’ grape cultivars. The heatmap (**B**) is based on the average values of each individual compound, with numerical values representing the mean ± standard error. Letters indicate significant differences among cultivars, determined using one-way ANOVA and Tukey’s HSD test (lowercase letters) or LSD test (uppercase letters) at a 95% confidence interval. Abbreviations: der = derivative.

In the stilbene group (STB), nine representatives were analysed in grape berries, namely piceid and eight resveratrol derivatives (Figure 4B). In the ‘Bronner‘ cultivar, the main stilbenes were resveratrol hexoside 1 and 5, representing 93% of the total stilbene content. In the ‘Morava‘ cultivar, the most abundant was resveratrol hexoside 1, accounting for 61% of the total stilbene content, while in the ‘Muscaris’ cultivar, the main stilbene was resveratrol hexoside 5 (90% of total STB content). The highest stilbene content was typically found in Bronner grapes (0.58 mg/kg FW) and the lowest in ‘Morava’ grapes (0.17 mg/kg FW) (Figure 4A). Stilbenes are known to have antifungal properties. Increased biosynthesis of these compounds can be influenced by inducing genes that encode enzymes in the phenylpropanoid pathway. Some chemicals used for this purpose are metallic salts and benzothiadiazoles [66,67]. The reason some oligomeric stilbenes have antifungal activity is their hydrophobic properties and their ability to pass through the cell membrane [68].

**Figure 5:**
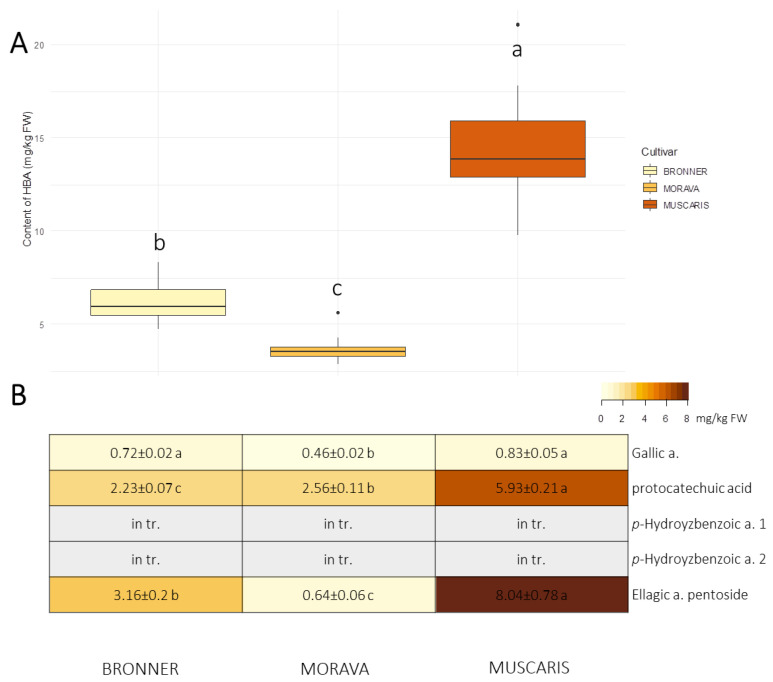
Total (**A**) and individual (**B**) hydroxybenzoic acid (HBA) content (mg/kg FW) in ‘Bronner’, ‘Morava’ and ‘Muscaris’ grape cultivars. The heatmap (**B**) is based on the average values of each individual compound, with numerical values representing the mean ± standard error. Letters indicate significant differences among cultivars, determined using one-way ANOVA and Tukey’s HSD test at a 95% confidence interval. Abbreviations: a = acid.

Among the hydroxybenzoic acids, gallic acid, protocatechuic acid, and ellagic acid pentoside were identified in the grapes of the studied cultivars, along with two p-hydroxybenzoic acids, which were present only in trace amounts (Figure 5B). The highest content of hydroxybenzoic acids was analysed in ‘Muscaris’ grape (Figure 5A). Martins et al. (2025) [69] reported that grape skins are a rich source of hydroxybenzoic acids (protocatechuic acid, gallic acid, syringic acid, and hydroxybenzoic acid), which are present in the skin in a bound form. Hydroxybenzoic acids are more stable to degradation and browning reactions under oxidative conditions compared to hydroxycinnamic acids, as their degradation impairs the organoleptic quality of musts and white wines [64]. The composition of phenolic compounds is also influenced by malolactic fermentation. During this process, lactic acid bacteria convert malic acid into lactic acid, reducing wine acidity and potentially modifying certain polyphenols depending on their chemical structure. For example, anthocyanins may lose colour as a result of pH changes occurring during fermentation [25].

The phenolic composition of grapes strongly influences the sensory properties, oxidative stability, and ageing potential of white wines. During winemaking, processes such as maceration, fermentation, oxygen exposure, and ageing influence the extraction and transformation of flavonoids, tannins, and other phenolic compounds. These compounds contribute to wine colour stability and antioxidant protection, and significantly influence the sensory characteristics of wine, including bitterness and astringency. Clarification processes help reduce excessive astringency and remove unstable phenolic substances, thereby improving wine clarity and overall balance [25,70].

In this study, the markedly higher concentration of flavan-3-ols in ‘Muscaris’ and ‘Bronner’ compared with ‘Morava’ suggests that wines from these cultivars are likely to exhibit improved mouthfeel and structure, and perceptible bitterness or slight astringency, as catechin, epicatechin, and procyanidins are known to contribute to tactile sensations such as body, bitterness, and tannic grip in white wines (Samoticha et al., 2017 [52]; Jara-Palacios et al., 2014, [54]). The elevated caftaric acid levels in ‘Muscaris’ and ‘Bronner’ indicate a higher susceptibility of their musts to enzymatic browning during processing, which requires careful oxidative management in the winery (Bustamante et al., 2025, [64]). Conversely, ‘Morava’ has a higher flavonol content, dominated by quercetin glycosides. This is more associated with photoprotective roles in the berry than with direct sensory impact. This aligns with the expectation of lighter-bodied, fresher, and more aromatic wines that are less bitter and astringent. Polyphenolics also influence wine stability through oxidative processes [25]. The higher stilbene content in ‘Bronner’, particularly resveratrol hexosides, may indirectly benefit wine stability due to their antioxidant and antimicrobial properties, supporting better resistance to oxidative and microbial spoilage during vinification (Viret et al., 2018, [65]; Jeandet et al., 2023, [68]). Overall, the phenolic profiles suggest that ‘Muscaris’ and ‘Bronner’ are better-suited to producing more structured white wines or styles involving extended lees contact. In contrast, ‘Morava’ is more suitable for producing fresh, aromatic wines that are ready to drink early, with minimal phenolic extraction.

## 3. Material and Methods

### 3.1. Plant Material and Growth Conditions

In the present study, conducted in 2024, grapes from three white ‘Muscaris’, ‘Bronner’, and ‘Morava’ grapevine interspecific hybrid cultivars were studied. ‘Muscaris’ is a PIWI cultivar that is already quite widespread in some European countries (e.g., 114 ha in Germany) [71], while ‘Bronner’ and ‘Morava’ are less known and are being introduced in several countries. Data on the origin of the cultivars (Table 3) were taken from the Vitis International Cultivar Catalogue [72].

**Figure 6:**
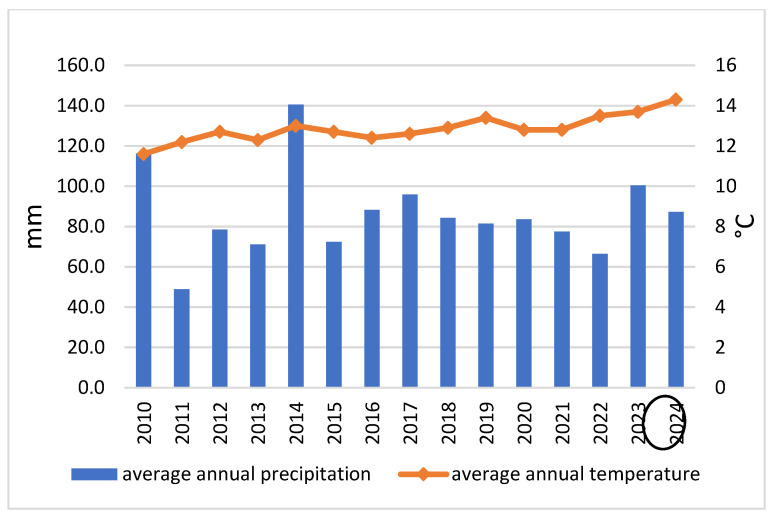
Average annual precipitations and average annual temperatures for Banja Luka in the period from 2010 to 2024.

The grapes of the analysed cultivars were produced in a vineyard located in the north-western part of the Republic of Srpska, in the city of Banja Luka (coordinates: 44.766667 °N, 17.183333 °E), Bosnia and Herzegovina. There are some differences in climate across the territory of Republika Srpska. The wider Banja Luka area (Northern Peri-Pannonian region) is characterised by moderately cold winters and warm summers. Precipitation decreases from west to east but is well distributed throughout the year. The absolute maximum air temperature is 41 °C, while the absolute minimum is −30 °C, indicating a high annual temperature amplitude, with the highest value being 71 °C. This region receives around 1900 h of sunshine per year [73]. Based on the values of average annual air temperatures and average annual precipitation in the Banja Luka area (Figure 6), recorded over the past 15 years (2010–2024), there is a clear trend of increasing average annual air temperatures since 2010, with the maximum value recorded in 2024 (14.3 °C). Regarding precipitation, excluding years with higher average levels (2010, 2014, and 2023), the average level in 2024 (87.2 mm) was within the range observed in most years of the period (71.0–88.2 mm).

**Figure 7:**
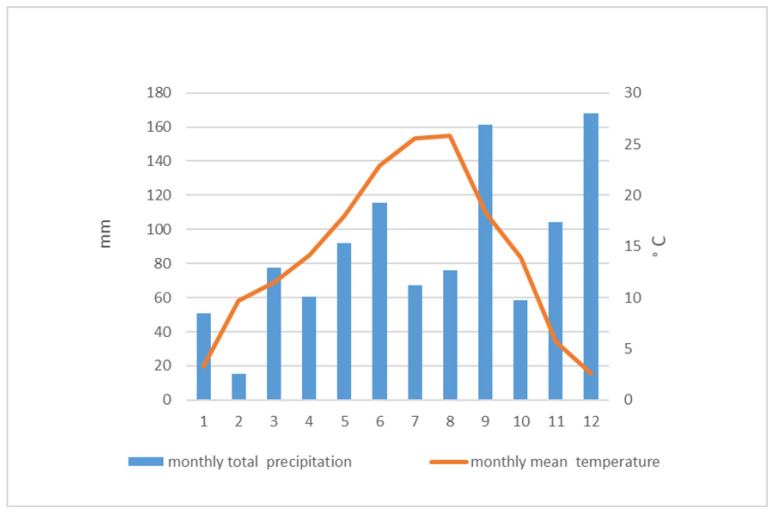
Monthly total precipitation and monthly mean temperatures for Banja Luka in 2024.

Given the importance of meteorological conditions during the vintage on grape quality, the average monthly temperatures and total monthly precipitation for the research year 2024 are also provided (Figure 7).

Based on the given data, significant differences were observed in precipitation levels and temperatures during the period of intense growth and ripening of grapes (July: 67.3 mm, 25.6 °C; August: 76.1 mm, 25.8 °C) compared to the harvest period (September: 161.3 mm, 18.3 °C).

### 3.2. Experimental Site and Grape Sampling

Grapes from all tested cultivars were harvested at technological maturity between 1 and 15 September 2024, from grapevines cultivated in four blocks, each consisting of ten vines. The planting distance was 2.0 m × 1.0 m, with vines trained to a single Royat cordon. The vineyard was managed according to the integrated concept of agriculture. Identical cultural practices were applied to all analysed cultivars throughout the growing season, without irrigation. Chemical protection of the vines and grapes included five treatments in accordance with the phenophases, using the following chemical preparations: Bordeaux mixture 20 WG, Nordox, Thiovit Jet, Delan Pro, Quadris, Switch 62.5 WG, and Dynali.

Grape sampling was conducted identically for each cultivar. For morphological and structural characteristics of the clusters and berries, five clusters per block were randomly selected (a total of 20 clusters per cultivar), and 10 berries from each cluster (a total of 200 berries per cultivar). For berry skin colour analysis, ten berries from each cluster were also used. For the determination of sugars, organic acids, and phenolic profile, four clusters per block (a total of 16 clusters per cultivar) were randomly selected. After transport to the laboratory, all measurements and extractions were performed.

### 3.3. Morphological and Structural Characterisation of the Bunch and Berries

Analysis of the morphological and structural properties of bunches and berries was carried out immediately after the samples were transported to the laboratory. The length and width of the berries were measured with a digital precision calliper, accuracy: 0.01 mm (UNIOR-270A, Zreče, Slovenia), and the mass of bunches and berries was measured with a precision balance (KERN-440, Balingen, Germany).

The berries selected for physical laboratory analysis were sampled from different parts of the bunch. The mass, length and width of the cluster, the number of berries per cluster, the grape and stem mass, and other physical parameters presented in Table 3 were determined for all cultivars studied. The uvological indices analysed included: the relative proportions of stem and berries in the cluster, the relative proportions of skin, seed and flesh per berry, the mass of seed and skin of 10 berries, the ratio of berry length to berry width, etc. (Table 3).

### 3.4. Determination of Berry Skin Colour and Total Soluble Content

The colour of the berry skin was determined by measuring a three-dimensional colour space values (L* for lightness, a* for red-greenness, and b* for blue-yellowness) on 10 berries in five repetitions for each cultivar, using a Minolta CR-300 Chroma handheld colorimeter (Minolta, Osaka, Japan). The CIRG index was calculated from the obtained measurements. The total soluble solid content was measured with a digital refractometer (ATAGO PAL BX/ACID, Japan), and the results are expressed in °Brix.

### 3.5. Determination of Sugars and Organic Acids Contents

The extract used for the determination of sugars and organic acids content in berries was obtained as follows: the berries from each cluster were homogenised in a blender (Gorenje, BN1000W VitaWAY, Velenje, Slovenia). Approximately 5.0 g of this homogenate was weighed using analytical balance (KERN & Sohn GmbH, ABJ 120-4M, Balingen, Germany), and the exact mass was recorded. The homogenate was then mixed with 25 mL of bi-distilled water, and the samples were shaken for 45 min at room temperature using an orbital shaker (Vise Shake, SHR 1D, Wertheim, Germany). The samples were then centrifuged in a laboratory centrifuge (Domel, CENTRIC 322 A, Železniki, Slovenia) at 6000 rpm for 10 min. Five millilitres of the resulting supernatant were transferred to Eppendorf tubes and stored at −20 °C until high-performance liquid chromatography (HPLC) analysis.

Prior to HPLC analysis, the extracts were filtered through a 0.20 µm cellulose ester filter (Macherey–Nagel, Düren, Germany) and transferred into glass vials. The samples were analysed by HPLC (Thermo Scientific, San Jose, CA, USA). Sugars were determined using a Rezex RCM-monosaccharides Ca^2+^ (2%) column (Phenomenex, Torrance, CA, USA) heated to 65 °C with a refractive index detector. The mobile phase was double-distilled water, and the flow rate was 0.6 mL/min. Organic acids were analysed on the same HPLC system equipped with a UV detector (210 nm) and a Rezex ROA—organic acids H^+^ (8%) column, heated to 65 °C. The mobile phase was 4 mM sulphuric acid, with a flow rate of 0.6 mL/min. Sugars and organic acids were identified by comparing their retention times with standards. Their contents were expressed in g/kg fresh grape weight [74].

### 3.6. Determination of Phenolic Compounds

Five grams of homogenised grape berries were mixed with 7.0 mL of an extraction solution (80% methanol: 17% distilled water: 3% formic acid) and transferred to a cooled ultrasonic bath for 1 h (ISO LAB, Laborgeräte GmbH, Eschau, Germany). After extraction, samples were centrifuged at 10,000 rpm for 10 min, and 5.0 mL of supernatant was transferred to Eppendorf tubes and stored at −20 °C until analysis. The extracts were filtered through 0.20 μm PTFE filters (Macherey–Nagel) into vials before analysis. The methanol extracts were then analysed for phenolic compounds by HPLC.

The analysis of phenolic substances was carried out using HPLC (Thermo Scientific, San Jose, CA, USA) equipped with a photodiode array (PDA) detector set to two wavelengths (280 nm and 350 nm). Column Gemini C18 was used for the separation (Phenomenex). The column temperature was maintained at 25 °C. For each analysis, 20 μL of each extract was used. Two mobile phases were used: mobile phase A—96.9% water, 3% acetonitrile, and 0.1% formic acid; and mobile phase B—97% acetonitrile, 3% water, and 0.1% formic acid. These were mixed according to the gradient method described by Mikulic-Petkovsek, Koron and Rusjan [74]. The flow rate of the mobile phase was 0.6 mL/min, and the analysis time for each sample was 50 min.

The identification of phenolic compounds was performed using a mass spectrometer (LTQ XL Linear Ion Trap Mass Spectrometer, Thermo Fisher Scientific, Waltham, MA, USA) with electrospray ionisation (ESI) in the negative range and MSn scanning from *m*/*z* 115 to 1900, as described by Mikulic-Petkovsek, Koron and Rusjan [74]. The identification of phenolic compounds was established based on their retention times and their PDA spectra compared to standards and on the basis of the fragmentation patterns in different MS^n^ modes, which were compared with literature data. The content of phenolic compounds was calculated using the appropriate standard (Supplementary Table S2) and reported as mg/kg fresh weight of grapes.

### 3.7. Statistical Analysis

Statistical analyses were performed using R-Commander (R Foundation for Statistical Computing, Auckland, New Zealand, 2021). Data visualisation and statistical tests were conducted with the ggplot2 (v3.4.1) [75] and agricolae (v1.3.5) [76] packages. To assess differences among grape cultivars, one-way ANOVA was applied. Residual analysis ensured that ANOVA assumptions were met, with outliers identified using box plots and the identify_outliers function from the rstatix package (v0.7.2) [77]. Normality was tested with the Shapiro–Wilk test, while Levene’s test assessed homogeneity of variance. When significant differences were found, Tukey’s HSD or LSD test (*p* < 0.05) was used for multiple comparisons. If assumptions were violated, data were either log-transformed or analysed using the Kruskal–Wallis rank-sum test, followed by Dunn’s test with Holm’s correction for multiple comparisons.

## 4. Conclusions

In recent years, significant progress has been made in grapevine breeding and selection programmes aimed at increasing resistance to fungi, resulting in the development of new tolerant cultivars with important agronomic and oenological traits. Using these cultivars reduces the need for plant protection products and decreases the environmental impact of viticulture. However, wines produced from these cultivars remain insufficiently accepted and recognised by both producers and consumers, highlighting the need for a comprehensive evaluation of grape and wine quality attributes.

Therefore, the aim of this study was to evaluate the main quality parameters of three resistant interspecific grapevine cultivars: ‘Bronner’, ‘Muscaris’, and ‘Morava’. The results demonstrate that these cultivars produced high-quality bunches and berries and exhibited favourable physicochemical compositions. All studied interspecific tolerant cultivars have good grape quality and are therefore suitable for producing high-quality wines. ‘Muscaris’ had the highest total sugar content, allowing a higher alcohol level in the wine. The highest phenolic content was found in ‘Bronner’ and ‘Muscaris’ grapes, which may give the wine a more intense flavour. ‘Bronner’ must had the highest titratable acid content, making it more suitable for wines where higher acidity is desired.

Given the high quality of the grapes, the cultivars studied can be recommended for cultivation in vineyards using organic production methods. Future studies should focus on the metabolic pathways responsible for phenolic accumulation in interspecific cultivars, as well as on how genotype–environment interactions affect phenolic composition and wine aroma expression.

## Figures and Tables

**Table 1:** Comparison of berry, cluster, and must chemical and physical quality parameters among ‘Bronner’, ‘Morava’ and ‘Muscaris’ grape cultivars.

Berry Quality Parameter	‘Bronner’	‘Morava’	‘Muscaris’
Lightness (L*)	32.38 ± 0.22 C	37.32 ± 0.24 A	34.74 ± 0.2 B
a* parameter	−0.58 ± 0.09	−1.28 ± 0.09	−2.4 ± 0.08
b* parameter	9.54 ± 0.18 b	5.62 ± 0.18 c	12.27 ± 0.21 a
Chroma (C*)	9.66 ± 0.18 b	5.96 ± 0.17 c	12.61 ± 0.2 a
Hue angle (°)	94.75 ± 0.64 B	106.71 ± 1.19 A	102.35 ± 0.59 A
CIRG index	2.04 ± 0.02 A	1.71 ± 0.03 B	1.64 ± 0.01 C
Total soluble solids (°Brix)	22.32 ± 0.17	22.03 ± 0.36	23.55 ± 0.56
Total titratable acids (TA g/L)	7.35 ± 0.08 a	6.4 ± 0.07 b	5.56 ± 0.09 c
pH	3.35 ± 0.01 C	3.53 ± 0.02 B	3.66 ± 0.04 A
Cluster weight (g)	281.5 ± 10.20 a	177.5 ± 7.50 b	254.0 ± 9.53 a
Cluster length (cm)	15.73 ± 0.41 a	14.24 ± 0.36 b	16.14 ± 0.4 a
Cluster width (cm)	10.77 ± 0.37	11.06 ± 0.28	10.27 ± 0.35
Grape stem mass (g)	8.76 ± 0.55 b	7.11 ± 0.34 c	10.4 ± 0.65 a
Number of berries per cluster	162.05 ± 6.34 a	120.05 ± 5.62 b	148.45 ± 6.11 a
Weight of berries per cluster (g)	272.77 ± 9.77 a	170.38 ± 7.33 b	243.62 ± 9.1 a
Relative proportions of stem (%)	3.09 ± 0.12 b	4.04 ± 0.14 a	4.08 ± 0.17 a
Relative proportions of berries (%)	96.91 ± 0.12 a	95.96 ± 0.14 b	95.92 ± 0.17 b
Mass of 10 berries (g)	17.31 ± 0.28	16.36 ± 0.35	16.82 ± 0.47
Average berry length of 10 berries (mm)	13.86 ± 0.27 a	13 ± 0.15 b	12.77 ± 0.17 b
Average berry width of 10 berries (mm)	12.96 ± 0.23	12.98 ± 0.21	12.42 ± 0.17
Berry length/berry width	1.07 ± 0.01 a	0.98 ± 0 c	1.03 ± 0.01 b
Skin mass of 10 berries (g)	1.84 ± 0.07 a	1.64 ± 0.06 b	1.78 ± 0.06 ab
Seed mass of 10 berries (g)	1 ± 0.02	0.97 ± 0.03	1.03 ± 0.04
Number of seeds per 10 berries	28.8 ± 0.68 a	22.55 ± 0.74 c	25.8 ± 0.9 b
Flesh mass of 10 berries (g)	14.47 ± 0.27	13.76 ± 0.32	14.02 ± 0.42
Relative proportions of skin (%)	10.7 ± 0.41	10.03 ± 0.29	10.33 ± 0.22
Relative proportions of seed (%)	5.76 ± 0.11	5.94 ± 0.22	6.13 ± 0.2
Relative proportions of flesh (%)	83.55 ± 0.44	84.03 ± 0.34	83.53 ± 0.17

Data are presented as the mean ± standard error. Significant differences among grape cultivars are indicated by letters, determined using one-way ANOVA and Tukey’s multiple comparison test at a 95% confidence interval (lowercase letters). When the assumptions for one-way ANOVA were not met, the Kruskal–Wallis test followed by Dunn’s multiple comparisons test with Holm’s correction for *p*-values was used (uppercase letters). Abbreviation: TA—tartaric acid.

**Table 2:** Comparison in the content of primary metabolites (g/kg FW) among ‘Bronner’, ‘Morava’ and ‘Muscaris’ grape cultivars.

	‘Bronner’	‘Morava’	‘Muscaris’
Sucrose	10.74 ± 0.12 b	12.58 ± 0.26 a	13.34 ± 0.29 a
Glucose	76.83 ± 0.74 b	72.11 ± 1.69 b	85.18 ± 2.05 a
Fructose	74.32 ± 0.71 B	68.5 ± 1.47 C	83.93 ± 1.94 A
Total sugars	161.89 ± 1.38 B	153.19 ± 2.96 C	182.46 ± 4.09 A
Citric	1.34 ± 0.07 c	1.69 ± 0.06 b	2.31 ± 0.12 a
Tartaric	7.24 ± 0.10	7.55 ± 0.15	7.09 ± 0.17
Malic	3.12 ± 0.11 c	3.93 ± 0.15 b	5.97 ± 0.22 a
Shikimic	0.01 ± 0.00 b	0.02 ± 0.00 a	0.01 ± 0.00 b
Fumaric	0.01 ± 0.00 b	0.01 ± 0.00 c	0.02 ± 0.00 a
Total organic acids	11.73 ± 0.22 c	13.2 ± 0.27 b	15.4 ± 0.37 a
S/A ratio	13.87 ± 0.29 a	11.71 ± 0.39 b	11.9 ± 0.27 b

Data are presented as the mean ± standard error. Significant differences among grape cultivars are indicated by letters, determined using one-way ANOVA and Tukey’s multiple comparison test at a 95% confidence interval (lowercase letters). When the assumptions for one-way ANOVA were not met, the Kruskal–Wallis test, followed by Dunn’s multiple comparisons test with Holm’s correction for *p*-values, was used (uppercase letters). Abbreviation: S/A—sugars/organic acids.

**Table 3:** Origin and pedigree of the studied interspecific hybrids hybrid cultivars.

Cultivar	Origin of the Cultivar	Pedigree	Year Crossing
‘Muscaris’	Germany	Solaris × Gelber Muscateller	1987
‘Bronner’	Germany	Merzling × Geisenheim 6494	1975
‘Morava’	Serbia	((Kunbarat × Tramin) × Bianca) × Rajnai Rizling GM 239-20	No data

## Data Availability

The data presented in this study are available in the article.

## Supplementary Materials

The following supporting information can be downloaded at: https://www.mdpi.com/article/10.3390/plants15111663/s1, Table S1. Identification of phenolic compounds in grape berries of interspecific hybrid cultivars in negative ionisation with HPLC-MS. Table S2. The list of phenolic standards and their calibration equations. Figure S1. Chromatogram of ‘Bronner’ cultivar recorded at 280 nm. Peak numbers are described in Table S1. Figure S2. Chromatogram of ‘Bronner’ cultivar recorded at 350 nm. Peak numbers are described in Table S1.
