# Supplementary material for: Comparative Evaluation and Physicochemical Characterisation of Three Tolerant Interspecific Grape Cultivars
Source: Plants (Basel). 2026 May 28;15(11):1663. doi: 10.3390/plants15111663 (PMC13259370; doi:10.3390/plants15111663)
Supplement: Supplementary file 1 [file plants-15-01663-s001.zip › Supplementary Table S1.pdf]

Supplementary Table S1: Identification of phenolic compounds in grape berries of interspecific hybrid cultivars in negative ionization with HPLC-MS

Peak Number	Rt	[M–H]–	Main MS/MS Fragments (m/z)	Phenolic compound	Bronner	Muscaris	Morava	Reference
1	6.16	169	125	Gallic acid	+	+	+	Goufo et al., 2020, Sukovic et al., 2020, Escobar-Avello et al., 2019
2	6.54	865	695, 577, 575, 289	Procyanidin trimer 1	+	+	+	Sukovic et al., 2020
3	7.56	153	123, 109	protocatechuic acid	+	+	+	Goufo et al., 2020, Sukovic et al., 2020, Escobar-Avello et al., 2019
4	8.70	305	179, 261, 221, 165, 121	Gallocatechin	+	+	+	Escobar-Avello et al., 2019
5	9.92	137	93, 109	*p*-Hydroxybenzoic acid 1	+	+	+	Goufo et al., 2020, Escobar-Avello et al., 2019
5	9.92	341	179, 161, 135	Caffeic acid derivative 1	+	+	+	Sukovic et al., 2020
6	10.93	577	425, 407, 451, 289	Procyanidin dimer 1	+	+	+	Sukovic et al., 2020
7	11.35	305	179, 261, 221, 165, 121	epigallocatechin 1	+	+	+	Escobar-Avello et al., 2019
8	11.57	577	393, 425, 439, 559, 269, 289	Procyanidin dimer 2	+	+	+	Sukovic et al., 2020
9	12.11	341	179, 135	Caffeic acid derivative 2	+	+	+	Sukovic et al., 2020
9	12.11	325	163, 119	Coumaric acid hexoside 1	+	+	+	Sukovic et al., 2020
10	12.13	341	179, 135	Caffeic acid derivative 3	+	+	+	Sukovic et al., 2020
10	12.13	577	425, 407, 451, 289	Procyanidin dimer 3	+	+	+	Sukovic et al., 2020
11	12.67	865	695, 577, 575, 289	Procyanidin trimer 2	+	+	+	Sukovic et al., 2020
12	12.93	311	149, 179	Caftaric acid	+	+	+	Sukovic et al., 2020
12	12.93	137	93, 109	*p*-Hydroxybenzoic acid 2	+	+	+	Goufo et al., 2020, Escobar-Avello et al., 2019
12	12.93	289	245, 205, 179	catechin	+	+	+	Goufo et al., 2020, Sukovic et al., 2020
12	12.93	577	425, 407, 451, 289	Procyanidin dimer 4	+	+	+	Sukovic et al., 2020
12	12.93	865	695, 577, 575, 289	Procyanidin trimer 3	+	+	+	Sukovic et al., 2020
13	13.41	865	695, 577, 575, 289	Procyanidin trimer 4	+	+	+	Sukovic et al., 2020
13	13.41	1153	1153, 575, 577, 865	Procyanidin tetramer 1	+	+	+	Prodanov et al., 2013
14	13.89	341	179, 135	Caffeic acid derivative 4	+	+	+	Sukovic et al., 2020
14	13.89	577	425, 407, 451, 289	Procyanidin dimer 5	+	+	+	Sukovic et al., 2020

15	14.18	577	425, 407, 451, 289	Procyanidin dimer 6	+	+	+	Sukovic et al., 2020
16	14.63	305	261, 199, 175	epigallocatechin 2	+	+	-	Escobar-Avello et al., 2019
16	14.63	1153	1153, 575, 577, 865	Procyanidin tetramer 2	+	+	+	Prodanov et al., 2013
17	15.38	289	245, 205, 179	epicatechin	+	+	+	Goufo et al., 2020, Sukovic et al., 2020
17	15.38	179	135, 143, 161, 89	Caffeic acid	+	+	+	Goufo et al., 2020, Sukovic et al., 2020
18	16.10	163	119	*p*-Coumaric acid	+	+	+	Sukovic et al., 2020
18	16.10	295	149, 163, 119	Coutaric acid 1	+	+	+	Sukovic et al., 2020
19	16.49	865	695, 577, 575, 289	Procyanidin trimer 5	+	+	+	Sukovic et al., 2020
19	16.49	295	163, 149, 119	Coutaric acid 2	+	+	+	Sukovic et al., 2020
20	17.24	325	193, 149, 178	ferulic acid pentose	+	+	+	Goufo et al., 2020
21	17.65	325	193, 113, 131, 149	Fertaric acid	+	+	+	Sukovic et al., 2020
22	13.88	453	359, 265	resveratrol dimer	+	+	+	Escobar-Avello et al., 2019
23	14.36	389	371, 269, 227, 185, 251, 209, 143	Piceid	+	+	+	Sukovic et al., 2020
24	15.48	389	269, 343, 371	Resveratrol derivative 1	+	tr	+	Sukovic et al., 2020
25	17.83	639	477, 301, 463	Quercetin-3-glucosyl-glucuronide	+	+	+	Goufo et al., 2020
26	20.12	609	301	Quercetin-3-rutinoside	+	+	+	Goufo et al., 2020
27	20.93	463	301, 300	Quercetin-3-galactoside	+	+	+	Goufo et al., 2020
27	20.93	905	811, 717, 227	Resveratrol tetramer 1	+	+	+	Escobar-Avello et al., 2019
27	20.93	479	317	Myricetin hexoside 1	+	+	+	Sukovic et al., 2020
28	21.11	463	301, 301	Quercetin-3-glucoside	+	+	+	Goufo et al., 2020, Sukovic et al., 2020
28	21.11	905	811, 717, 227	Resveratrol tetramer 2	+	+	+	Escobar-Avello et al., 2019
29	21.55	593	285	Kaempferol-3-rutinoside	+	+	+	Goufo et al., 2020
30	21.69	389	227, 305, 175, 185	Resveratrol derivative 2	+	+	+	Sukovic et al., 2020
30	21.69	433	301, 300	Ellagic acid pentoside	+	+	+	Goufo et al., 2020, Escobar-Avello et al., 2019
30	21.69	623	315, 300	Isorhamnetin-3-rutinoside	+	+	+	Goufo et al., 2020
31	22.14	447	285, 327, 419, 255, 243, 151	Kaempferol-3-galactoside	+	+	+	Goufo et al., 2020
31	22.14	477	301, 179	Quercetin-3-glucuronide	+	+	+	Goufo et al., 2020
31	22.14	479	317	Myricetin hexoside 2	+	+	+	Sukovic et al., 2020
32	22.64	447	285, 255, 327, 151, 227	Kaempferol-3-glucoside	+	+	+	Goufo et al., 2020

32	22.64	389	227, 371, 343, 251	Resveratrol derivative 3	+	+	in tr.	Sukovic et al., 2020
32	22.64	447	301	Quercetin-3-rhamnoside	+	+	-	Goufo et al., 2020
33	22.80	477	301, 315, 314, 357, 151	Isorhamnetin-3-galactoside	+	+	+	Goufo et al., 2020
33	22.80	389	227, 305, 175, 185	Resveratrol derivative 4	+	+	in tr.	Sukovic et al., 2020
33	22.80	507	344, 387, 479	Syringetin hexoside	-	+	+	Sukovic et al., 2020
34	23.42	433	269, 287, 259, 151	Dihydrokaempferol-3-rhamnoside	+	+	+	Goufo et al., 2020
34	23.42	463	317	Myricetin-3-rhamnoside	+	+	-	Goufo et al., 2020
34	23.42	447	285	Kaempferol hexoside	+	+	+	Sukovic et al., 2020
35	23.65	389	227, 305, 175, 185	Resveratrol derivative 5	+	+	in tr.	Sukovic et al., 2020
