# Supplementary material for: Comparative Evaluation and Physicochemical Characterisation of Three Tolerant Interspecific Grape Cultivars
Source: Plants (Basel). 2026 May 28;15(11):1663. doi: 10.3390/plants15111663 (PMC13259370; doi:10.3390/plants15111663)
Supplement: Supplementary file 1 [file plants-15-01663-s001.zip › Supplementary Table S2.pdf]

Supplementary Table S2: The list of phenolic standards and their calibration equations.

Phenolic group	Phenolic compound	Standard for calculation	Regression equation	R^2	Supplier	Produc code
HBA	Gallic acid	Gallic acid	y=7717.5x	0.9972	Merck	1596300001
FLA	Procyanidin trimer 1	Procy B2	y=164.33x	0.9959	Sigma Aldrich	42157
HBA	protocatechuic acid	Protocateuchic acid	y=1370.5x	0.9964	Fluka	37580
FLA	Gallocatechin	(+) katehin hidrat	y=251.35x	0.9929	Fluka	22110
HBA	*p*-Hydroyzbenzoic acid 1	Gallic acid	y=7717.5x	0.9972	Merck	1596300001
HCA	Caffeic acid derivative 1	Caffeic acid	y=903.14x	0.9972	Sigma	C-0625
FLA	Procyanidin dimer 1	Procy B2	y=164.33x	0.9959	Sigma Aldrich	42157
FLA	epigallocatechin 1	(+) katehin hidrat	y=251.35x	0.9929	Fluka	22110
FLA	Procyanidin dimer 2	Procy B2	y=164.33x	0.9959	Sigma Aldrich	42157
HCA	Coumaric acid hexoside 1	*p*-coumaric acid	y=1742.52x	0.9999	Sigma Aldrich	C9008
HCA	Caffeic acid derivative 2	Caffeic acid	y=903.14x	0.9972	Sigma	C-0625
HCA	Caffeic acid derivative 3	Caffeic acid	y=903.14x	0.9972	Sigma	C-0625
FLA	Procyanidin dimer 3	Procy B2	y=164.33x	0.9959	Sigma Aldrich	42157
FLA	Procyanidin trimer 2	Procy B2	y=164.33x	0.9959	Sigma Aldrich	42157
HBA	*p*-Hydroyzbenzoic acid 2	Gallic acid	y=7717.5x	0.9972	Merck	1596300001
FLA	catechin	(+) katehin hidrat	y=251.35x	0.9929	Fluka	22110
FLA	Procyanidin dimer 4	Procy B2	y=164.33x	0.9959	Sigma Aldrich	42157
FLA	Procyanidin trimer 3	Procy B2	y=164.33x	0.9959	Sigma Aldrich	42157
HCA	Caffeic acid	Caffeic acid	y=903.14x	0.9972	Sigma	C-0625
HCA	Caftaric acid	Caffeic acid	y=903.14x	0.9972	Sigma	C-0625
FLA	Procyanidin trimer 4	Procy B2	y=164.33x	0.9959	Sigma Aldrich	42157
FLA	Procyanidin tetramer 1	Procy B2	y=164.33x	0.9959	Sigma Aldrich	42157
HCA	Caffeic acid derivative 4	Caffeic acid	y=903.14x	0.9972	Sigma	C-0625
FLA	Procyanidin dimer 5	Procy B2	y=164.33x	0.9959	Sigma Aldrich	42157
FLA	Procyanidin dimer 6	Procy B2	y=164.33x	0.9959	Sigma Aldrich	42157
FLA	epigallocatechin 2	(+) katehin hidrat	y=251.35x	0.9929	Fluka	22110

FLA	Procyanidin tetramer 2	Procy B2	y=164.33x	0.9959	Sigma Aldrich	42157
FLA	epicatechin	(-) Epicatechin	y=272.69x	0.9991	Fluka	45300
HCA	*p*-coumaric acid	*p*-coumaric acid	y=1742.52x	0.9999	Sigma Aldrich	C9008
HCA	Coutaric acid 1	*p*-coumaric acid	y=1742.52x	0.9999	Sigma Aldrich	C9008
FLA	Procyanidin trimer 5	Procy B2	y=164.33x	0.9959	Sigma Aldrich	42157
HCA	Coutaric acid 2	*p*-coumaric acid	y=1742.52x	0.9999	Sigma Aldrich	C9008
HCA	ferulic acid pentose	Ferulic acid	y=1819.6x	0.9978	Fluka	46280
HCA	fertaric acid	Ferulic acid	y=1819.6x	0.9978	Fluka	46280
STB	Resveratrol dimer	Resveratrol	y=3851.6x	0.9953	Sigma	R5010
STB	Piceid	Resveratrol	y=3851.6x	0.9953	Sigma	R5010
STB	Resveratrol derivative 1	Resveratrol	y=3851.6x	0.9953	Sigma	R5010
FLO	Quercetin-3-(glucosyl)-glucuronide	Q-3-β-D-glucoside	y=609.46x	0.9983	Bichemika Fluka	17793
FLO	Quercetin-3-rutinoside	Q-3-β-D-glucoside	y=609.46x	0.9983	Bichemika Fluka	17793
FLO	Quercetin-3-galactoside	Rutin trihydrate	y=434.88x	0.9983	Fluka	78095
STB	Resveratrol tetramer 1	Resveratrol	y=3851.6x	0.9953	Sigma	R5010
FLO	Myricetin hexoside 1	Myricetin 3-O-β-D-Galactopyranoside	y=496.61x	0.977	Sigma	SMB00249
FLO	Quercetin-3-glucoside	Q-3-β-D-glucoside	y=609.46x	0.9983	Bichemika Fluka	17793
STB	Resveratrol tetramer 2	Resveratrol	y=3851.6x	0.9953	Sigma	R5010
FLO	Kaempferol-3-rutinoside	Kaempferol-3-glucoside	y=1075.1x	0.9983	Sigma	79851
STB	Resveratrol derivative 2	Resveratrol	y=3851.6x	0.9953	Sigma	R5010
HBA	Ellagic acid pentoside	Ellagic acid	y=210.76x	0.9873	Sigma	E-2250
FLO	Isorhamnetin-3-rutinoside	Iso-3-rut	y=1611.1x	0.9807	Phytolab	83337
FLO	Kaempferol-3-galactoside	Kaempferol-3-glucoside	y=1075.1x	0.9983	Sigma	79851
FLO	Quercetin-3-glucuronide	Q-3-glucuronide	y=1138.6x	0.9972	Sigma	90733
FLO	Myricetin hexoside 2	Myricetin 3-O-β-D-Galactopyranoside	y=496.61x	0.977	Sigma	SMB00249
STB	Resveratrol derivative 3	Resveratrol	y=3851.6x	0.9953	Sigma	R5010
FLO	Kaempferol-3-glucoside	Kaempferol-3-glucoside	y=1075.1x	0.9983	Sigma	79851
FLO	Syringetin hexoside	Q-3-β-D-glucoside	y=609.46x	0.9983	Bichemika Fluka	17793
STB	Resveratrol derivative 4	Resveratrol	y=3851.6x	0.9953	Sigma	R5010

FLO	Quercetin-3-rhamnoside	Q-3-β-D-glucoside	y=609.46x	0.9983	Bichemika Fluka	17793
FLO	Isorhamnetin-3-galactoside	Iso-3-rut	y=479.68x	0.9807	Phytolab	83337
FLO	Dihydrokaempferol-3-rhamnoside	Kaempferol-3-glucoside	y=1075.1x	0.9983	Sigma	79851
FLO	Kaempferol-hexoside	Kaempferol-3-glucoside	y=1075.1x	0.9983	Sigma	79851
FLO	Myricetin-3-rhamnoside	Myricetin 3-O-β-D-Galactopyranoside	y=496.61x	0.977	Sigma	SMB00249
STB	Resveratrol derivative 5	Resveratrol	y=3851.6x	0.9953	Sigma	R5010
