# Supplementary figures and images for: Comparative Evaluation and Physicochemical Characterisation of Three Tolerant Interspecific Grape Cultivars
Source: Plants (Basel). 2026 May 28;15(11):1663. doi: 10.3390/plants15111663 (PMC13259370; doi:10.3390/plants15111663)

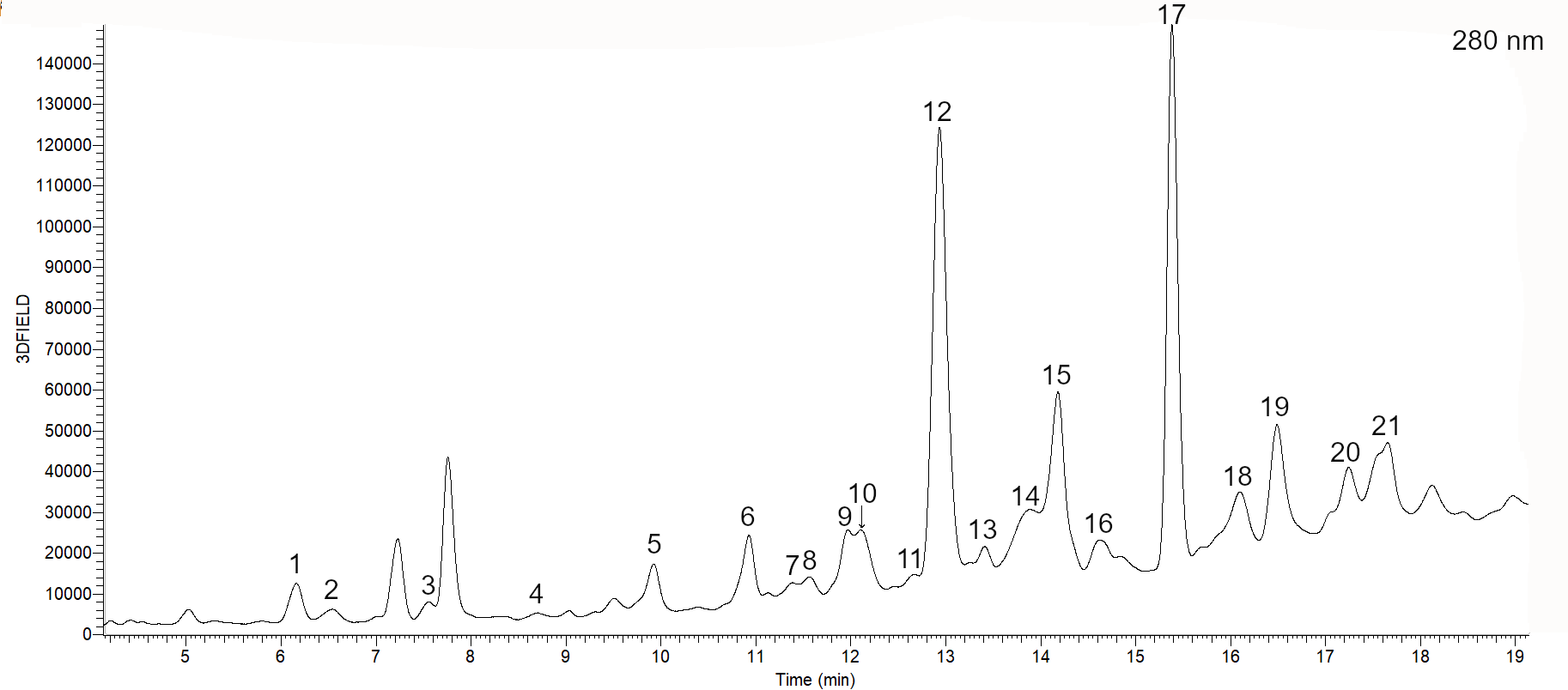

Supplement: Supplementary file 1 [file plants-15-01663-s001.zip › Supplementary Figure S1.png]

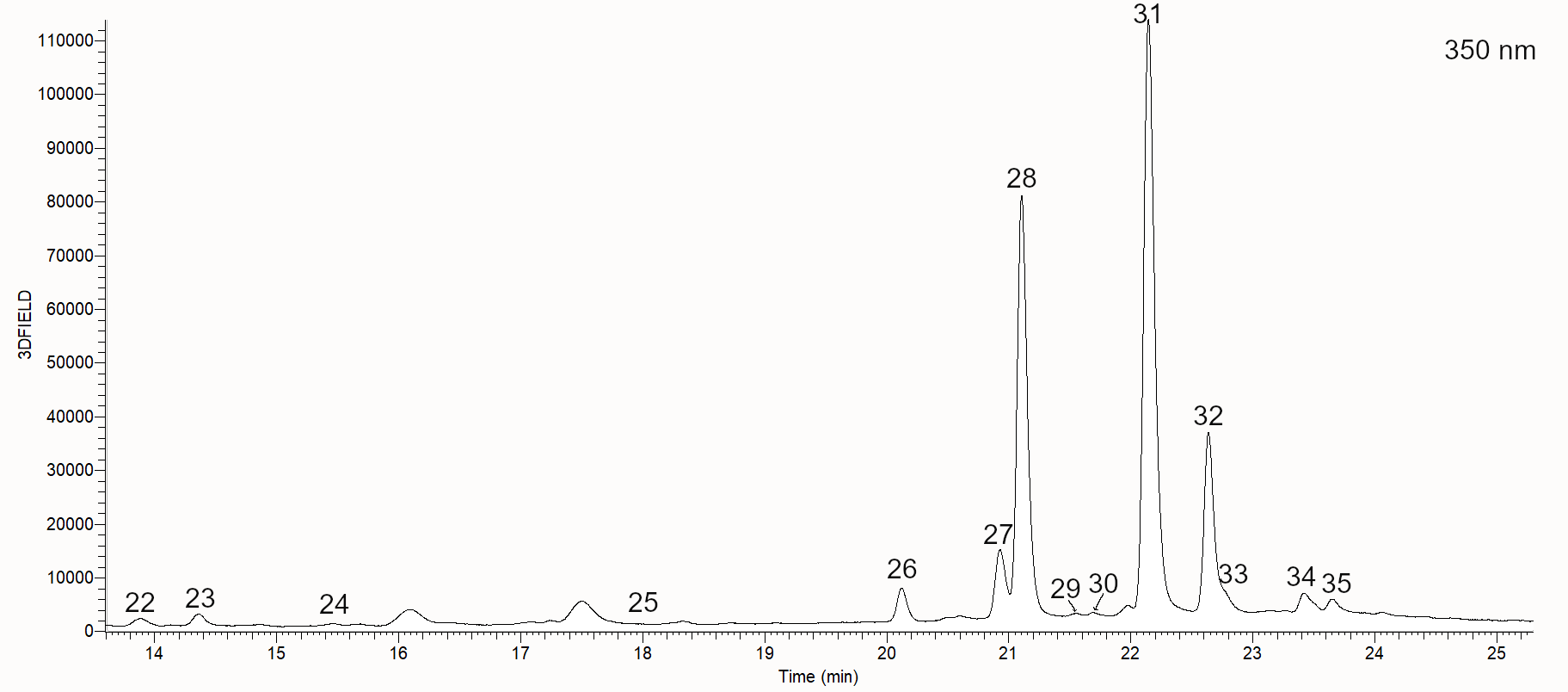

Supplement: Supplementary file 1 [file plants-15-01663-s001.zip › Supplementary Figure S2.png]
